# Supplementary material for: Alternaria alternata Isolated from Infected Pears (Pyrus communis) in Italy Produces Non-Host Toxins and Hydrolytic Enzymes as Infection Mechanisms and Exhibits Competitive Exclusion against Botrytis cinerea in Co-Infected Host Fruits
Source: J Fungi (Basel). 2023 Mar 7;9(3):326. doi: 10.3390/jof9030326 (PMC10053571; doi:10.3390/jof9030326)
Supplement: Supplementary file 1 [file jof-09-00326-s001.zip › jof-2146485-supplementary.pdf]

Supplementary Information

Figure S1. ^{1}H NMR spectrum (400 MHz) of altertoxin I (**1**) in $CDCl_3$ 2

Figure S2. ESI MS spectrum of altertoxin I (**1**) recorded in positive mode 2

Figure S3. ^{1}H NMR spectrum (400 MHz) of alteichin (**2**) in $CDCl_3$ 3

Figure S4. ESI MS spectrum of alteichin (**2**) recorded in positive mode 3

Figure S5. ^{1}H NMR spectrum (400 MHz) of alternariol (**3**) in CD_3OD 4

Figure S6. ESI MS spectrum of alternariol (**3**) recorded in positive mode 4

Figure S7. ^{1}H NMR spectrum (400 MHz) of alternariol 4-methyl ether (**4**) in $(CD_3)_2CO$ 5

Figure S8. ESI MS spectrum of alternariol 4-methyl ether (**4**) recorded in positive mode 5

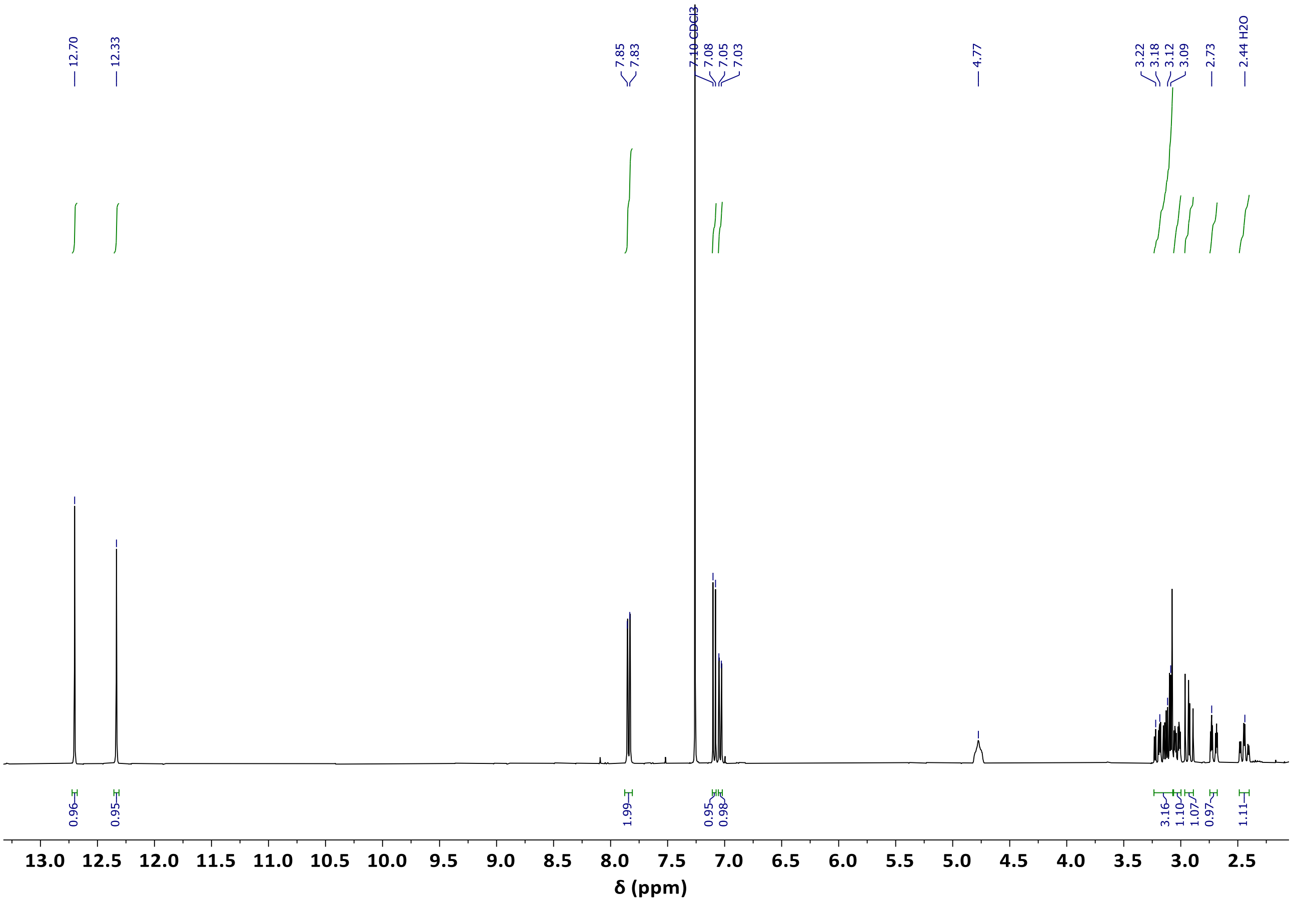

Figure S1. ^{1}H NMR spectrum (400 MHz) of altertoxin I (**1**) in $CDCl_3$

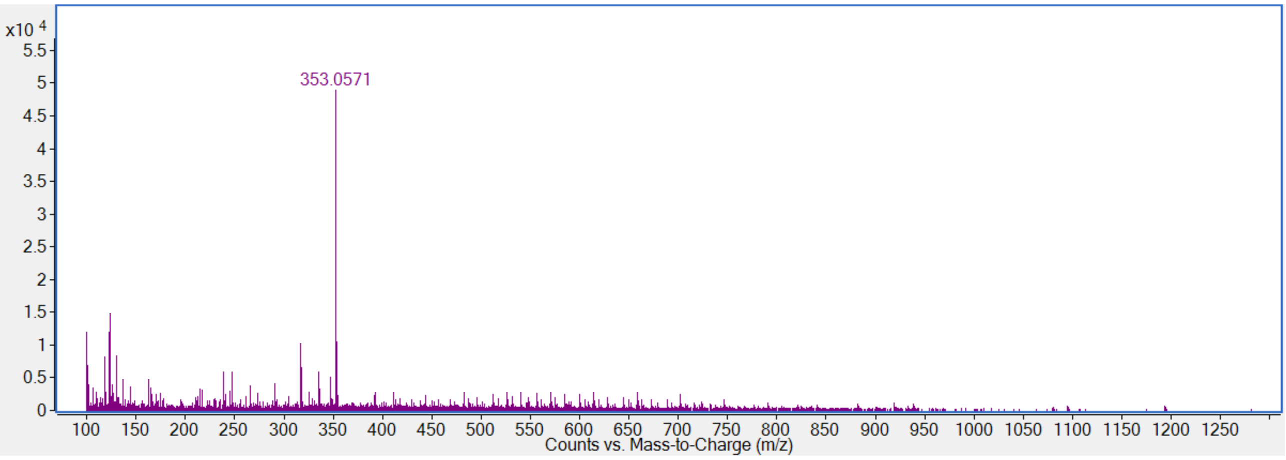

Figure S2. ESI MS spectrum of altertoxin I (**1**) recorded in positive mode

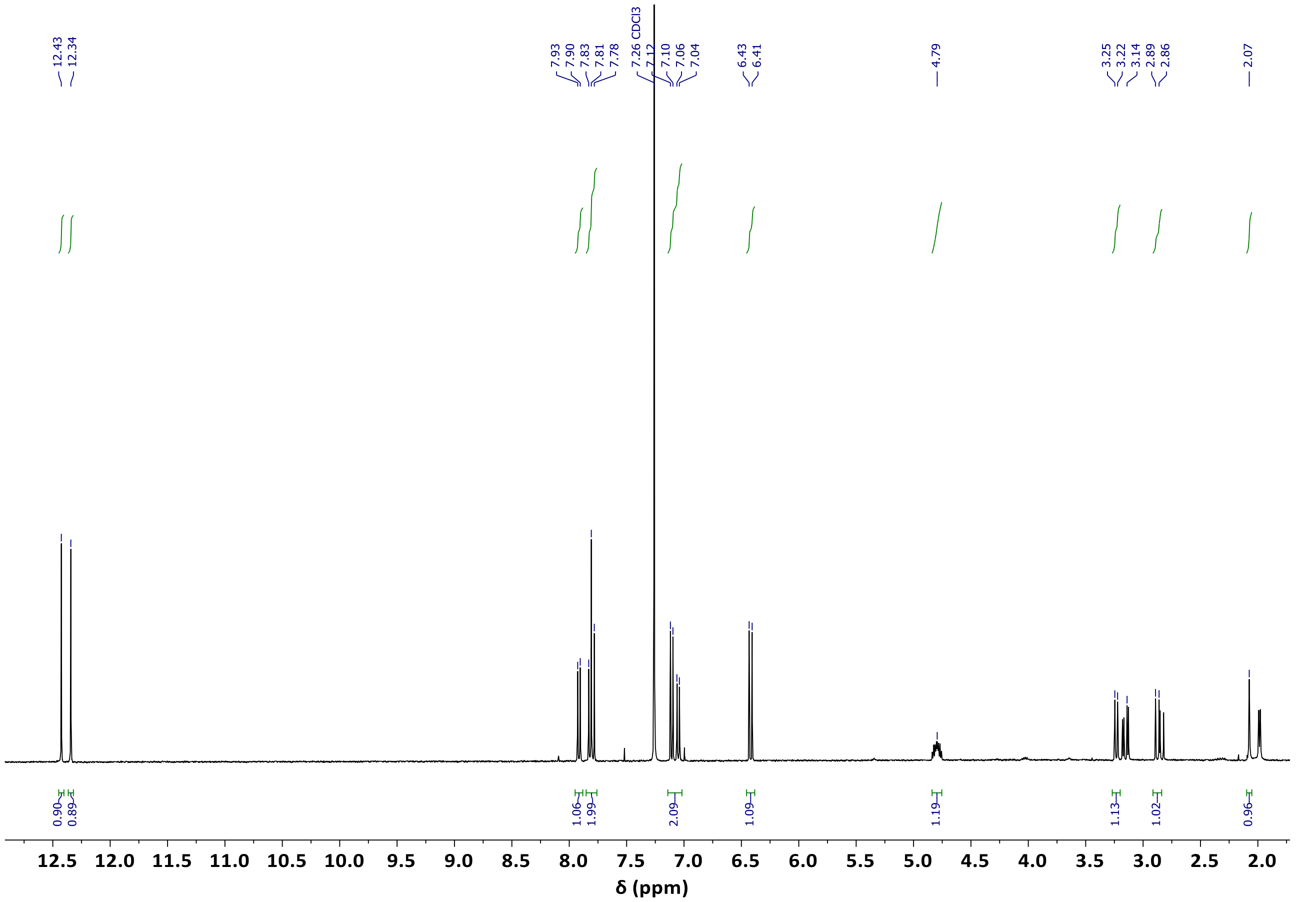

Figure S3. ^{1}H NMR spectrum (400 MHz) of alteichin (**2**) in $CDCl_3$

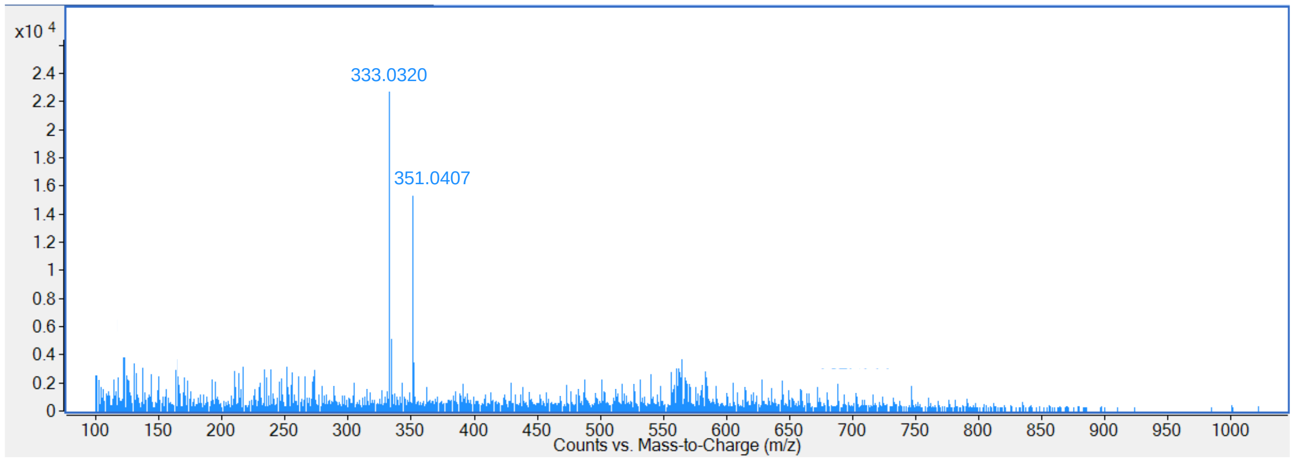

Figure S4. ESI MS spectrum of alteichin (**2**) recorded in positive mode

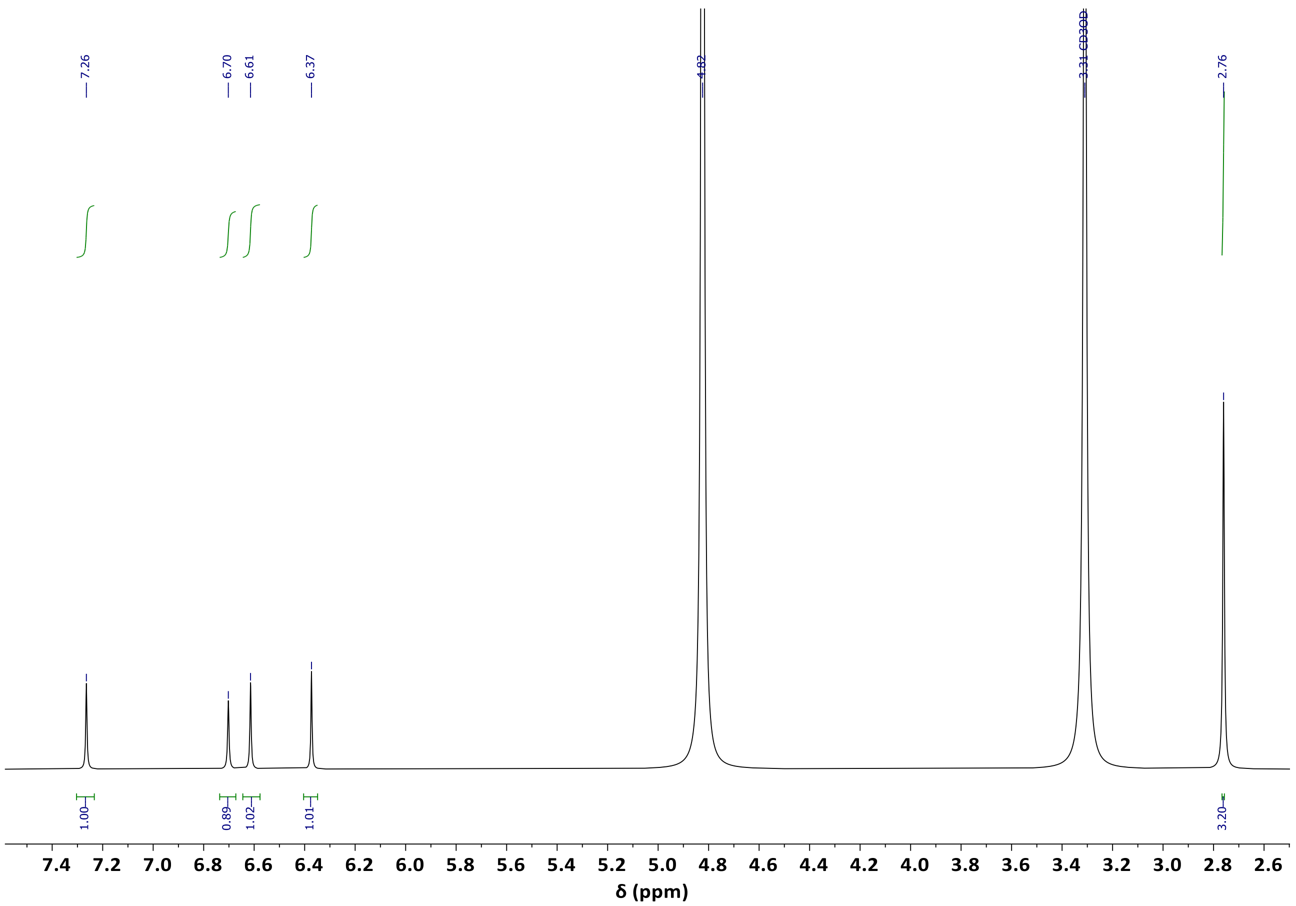

Figure S5. ^{1}H NMR spectrum (400 MHz) of alternariol (**3**) in CD_3OD

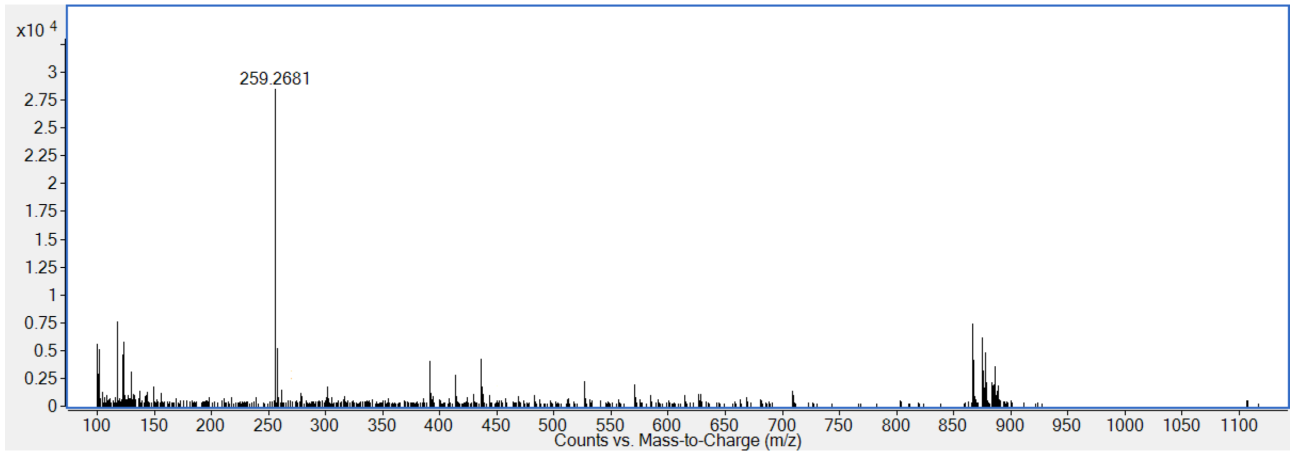

Figure S6. ESI MS spectrum of alternariol (**3**) recorded in positive mode

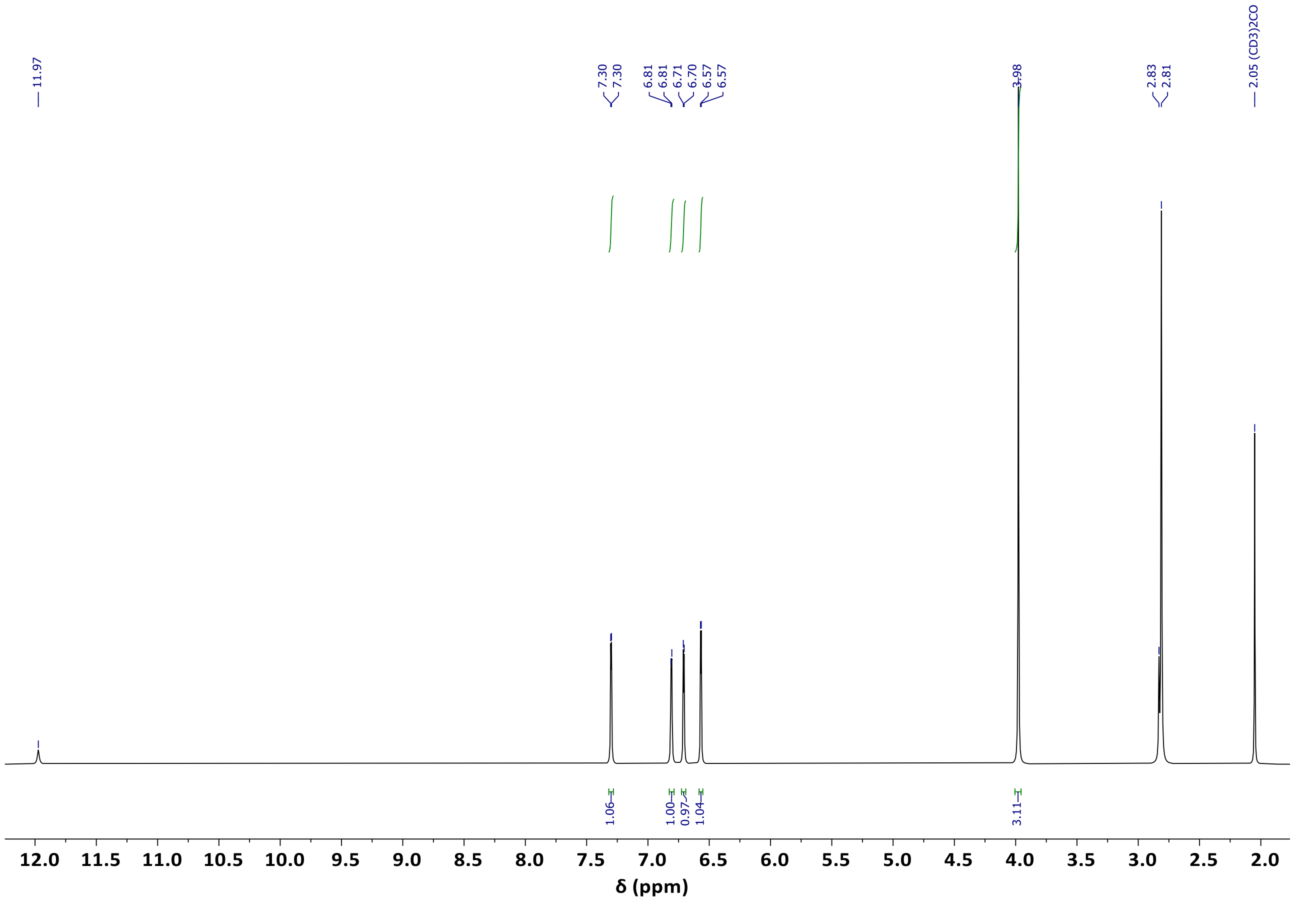

Figure S7. ^{1}H NMR spectrum (400 MHz) of alternariol 4-methyl ether (**4**) in $(CD_3)_2CO$

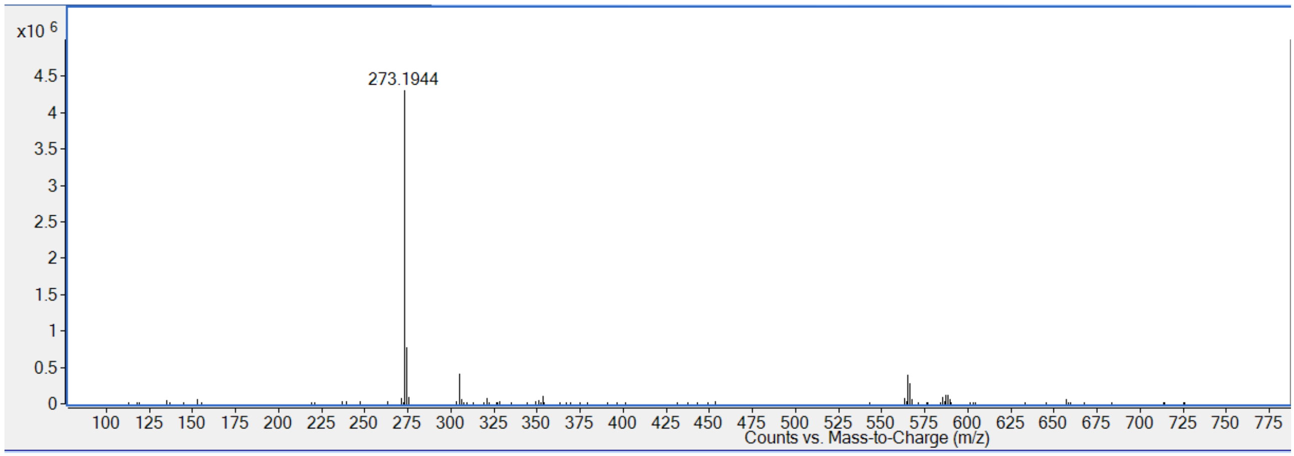

Figure S8. ESI MS spectrum of alternariol 4-methyl ether (**4**) recorded in positive mode
